# Effect of Combined Polyphenol on the Disease Course in Children Diagnosed with Influenza

**DOI:** 10.3390/pharmaceutics18070858

**Published:** 2026-07-14

**Authors:** Eren Güzeloğlu, Vefik Arica, Belen Ateş, Taner Adigüzel, Aysun Boğa, Ali Rıza Akgün, Mehmet Tolga Köle, Ayşe Ademoğlu, Hatice Demet Kemalbay, Feyza Aydın Özgür, Hatice Yıldız Özkan, Eda Çiftçi, Hüseyin Dağ

**Affiliations:** 1 Department of Pediatrics, Prof. Dr. Cemil Taşcıoğlu City Hospital, University of Health Sciences, Istanbul 34384, Türkiye; dr.erenguzeloglu@gmail.com (E.G.); belenterlemez@gmail.com (B.A.); ayademoglu@gmail.com (A.A.); hdkemalbay@gmail.com (H.D.K.); feyzozgr@gmail.com (F.A.Ö.); dr.haticeyildizozkan@hotmail.com (H.Y.Ö.); edacftc1@hotmail.com (E.Ç.); huseyindag2003@gmail.com (H.D.); 2 Department of Pediatrics, Yalova Training and Research Hospital, Faculty of Medicine, Yalova University, Yalova 77200, Türkiye; tanerdgzl@gmail.com; 3 Department of Pediatrics, Sancaktepe Prof. Dr. İlhan Varank Training and Research Hospital, University of Health Sciences, Istanbul 34785, Türkiye; aysunboga@gmail.com; 4 Department of Pediatrics, Esenyurt Necmi Kadıoğlu State Hospital, Istanbul 34517, Türkiye; alirizaakgun01@hotmail.com; 5 Department of Pediatrics, Ankara Etlik City Hospital, University of Health Sciences, Ankara 06170, Türkiye; mehmet_tolga@hotmail.com

**Keywords:** influenza, child, polyphenol, antiviral therapy

## Abstract

**Objective:** This multicenter retrospective real-world data study aimed to evaluate whether the use of a preparation containing polyphenol, vitamin D3, vitamin C, and zinc in addition to antiviral therapy was associated with symptom duration and severity, time to fever resolution, and functional recovery in children diagnosed with influenza. **Materials and Methods:** This was a five-center, multicenter, retrospective, comparative real-world data analysis. A total of 128 patients aged 4–10 years with a clinical diagnosis of influenza were classified into a control group receiving antiviral therapy alone (*n* = 64) and a combined polyphenol group receiving, in addition to antiviral therapy, a preparation containing polyphenol, vitamin D3, vitamin C, and zinc (*n* = 64). Clinical data were obtained from electronic patient records and parent-reported information. Symptom severity (0–3 ordinal score), recovery time, and functional outcomes were assessed on Days 0, 3, 5, and 7. Continuous variables were compared using the independent-samples *t* test, categorical variables using the chi-square or Fisher exact test, and changes over time using a repeated-measures general linear model. Statistical significance was accepted as a two-sided *p* value of <0.05. **Results:** In this study including 128 pediatric patients, there were no significant differences between the combined polyphenol and control groups in demographic or baseline clinical characteristics (*p* > 0.05). During follow-up, all symptom scores improved more rapidly and markedly in the combined polyphenol group than in the control group. By Day 3, significant differences were observed between groups in fever, cough, rhinorrhea, sore throat, fatigue, general condition, and physical activity scores (*p* < 0.001 for all parameters). By Day 5, almost complete resolution of symptoms (score 0) was observed in the combined polyphenol group, whereas symptoms persisted to a substantial extent in the control group (*p* < 0.001). The time to fever resolution was significantly shorter in the combined polyphenol group (1.84 ± 0.72 days vs. 4.5 ± 1.36 days; *p* < 0.001). Similarly, time to return to school (3.21 ± 0.9 days vs. 5.8 ± 1.91 days; *p* < 0.001) and time for parents to return to work were significantly shorter in the combined polyphenol group (*p* < 0.001). The need for additional healthcare visits and antipyretic use was markedly lower in the combined polyphenol group (Day 3: 20.3% vs. 53.1%; Day 5: 0% vs. 15.6%; *p* < 0.001 for both comparisons). Repeated-measures analyses showed a significant time-by-group interaction, indicating that the reduction in symptom scores occurred more rapidly and prominently in the combined polyphenol group (*p* < 0.001). Treatment was generally well tolerated in the combined polyphenol group; no serious adverse events were reported, and parent-reported tolerability was high (9.39 ± 0.7). **Conclusions:** This multicenter retrospective real-world data study suggests that the use of a preparation containing polyphenol, vitamin D3, vitamin C, and zinc in addition to antiviral therapy was associated with shorter symptom duration, lower symptom severity scores, and faster functional recovery in children diagnosed with influenza. These findings suggest that this combination may represent a promising and well-tolerated adjunctive approach in the management of pediatric influenza.

## 1. Introduction

Influenza is one of the most common acute respiratory tract infections in childhood and causes substantial morbidity and increased healthcare utilization every year. The disease, characterized by symptoms such as fever, cough, sore throat, and fatigue, is clinically important because of school absenteeism, parental work loss, and the risk of complications [1,2]. Antiviral treatments can reduce disease duration and the risk of complications, particularly when initiated within the first 48 h after symptom onset; however, in some cases, symptoms may be prolonged and the inflammatory response may persist despite such treatment. This has increased interest in supportive and adjuvant treatment approaches [3,4].

Polyphenols are biologically active compounds with antioxidant, anti-inflammatory, and antiviral properties that can modulate host responses during viral infections [5,6]. Micronutrients such as vitamin C, vitamin D3, and zinc are also involved in immune regulation and respiratory infection responses [5,7]. However, clinical data evaluating combined formulations containing these components in pediatric influenza remain limited.

From a pharmaceutical perspective, the evaluated preparation represents a standardized multi-component formulation combining pomegranate peel-derived polyphenols with vitamin D3, vitamin C, and zinc. Such formulations are of interest in pharmaceutics because they aim to deliver bioactive compounds with complementary antioxidant, anti-inflammatory, antiviral, and immunomodulatory properties within a single preparation. The standardization of polyphenol content and the combined delivery of micronutrients may be relevant for formulation development, therapeutic optimization, and real-world evaluation of adjunctive pharmaceutical products used in respiratory viral infections.

However, evidence regarding standardized multi-component polyphenol-containing formulations as adjunctive products in pediatric influenza remains limited, particularly in real-world multicenter settings. Most available data focus on individual micronutrients or in vitro antiviral mechanisms, whereas clinical data evaluating combined pharmaceutical preparations containing polyphenols, vitamin D3, vitamin C, and zinc are scarce. Therefore, real-world evidence may provide preliminary insight into the potential clinical relevance and tolerability of such formulations in routine pediatric practice.

This study aimed to evaluate the effects of a polyphenol-based combination therapy on the clinical course of children diagnosed with influenza using multicenter real-world data.

## 2. Materials and Methods

This was a multicenter, retrospective, observational, comparative real-world data study conducted in the pediatric outpatient clinics of five tertiary hospitals in Türkiye. The study included children diagnosed with influenza between 12 February 2026 and 12 April 2026, and retrospective clinical and follow-up data were extracted from electronic medical records and documented routine follow-up records. A total of 128 pediatric patients aged 4–10 years with a clinical and/or laboratory diagnosis of influenza were included.

Children aged 4–10 years were included because this age range represents the pediatric outpatient population in which the investigated formulation was most commonly used in routine practice and allows more reliable parent-supported symptom follow-up compared with younger children.

Patients were divided into two groups: a control group receiving standard antiviral therapy (oseltamivir phosphate) (*n* = 64) and an intervention group receiving, in addition to antiviral therapy, a combined polyphenol formulation (Ocean Polyphenols, Orzax Medicine Inc., Istanbul, Türkiye) containing standardized pomegranate peel-derived polyphenols (25 mg), vitamin D3 (400 IU), vitamin C (50 mg), and zinc (7.5 mg) per daily dose (*n* = 64).

As this was a retrospective real-world study, treatment allocation was not randomized. The decision to prescribe the polyphenol-containing preparation was made by the attending physicians according to routine clinical practice, individual clinical judgment, and family preferences when applicable. Patients were subsequently categorized into study groups based on the treatment documented in their medical records.

The decision to use the adjunctive formulation was made before retrospective data extraction and was independent of the present study.

No predefined randomization procedure, allocation algorithm, or study-specific treatment protocol was used. The decision to use the polyphenol-containing preparation was based on routine outpatient clinical practice and may have been influenced by physician preference, family acceptance, product availability, perceived symptom burden, and parental request.

Eligible patients were identified retrospectively from outpatient clinic records. A convenience sampling approach was used, and patients meeting the inclusion and exclusion criteria were included until equal group sizes were obtained for the control and combined formulation groups. No prospective sampling strategy was applied.

Inclusion criteria were as follows: age between 4 and 10 years; clinical and/or laboratory diagnosis of influenza A and/or B; receipt of oseltamivir therapy; availability of baseline symptom data; and documented follow-up information on at least Days 3 and 5.

Exclusion criteria were hospitalization at diagnosis, chronic systemic disease, immunodeficiency, chronic pulmonary disease, neurological disease affecting symptom assessment, incomplete medical records, missing follow-up data, bacterial coinfection requiring antibiotic therapy at baseline, and use of other immune-supportive supplements during the observation period.

Clinical data were obtained from patient files, outpatient clinic records, and parent feedback. Assessments were performed on the day of diagnosis (Day 0) and on Days 3, 5, and, where available, 7. Symptom severity was assessed using 0–3 ordinal scores for fever, cough, rhinorrhea, sore throat, and fatigue. General condition and physical activity level scores, time to fever resolution, time to return to school, time for parents to return to work, additional healthcare visits, and the need for additional treatments were also recorded. Adverse events and treatment-related tolerability were evaluated separately. Follow-up information was obtained from outpatient follow-up visits, electronic medical records, and documented parent feedback, including telephone-based follow-up when available. Therefore, not all Day 3, Day 5, and Day 7 assessments required an in-person healthcare visit.

### Symptom Scoring

Symptom severity was evaluated using a 0–3 ordinal scale: 0 = absent, 1 = mild, 2 = moderate, and 3 = severe. For fever, the ordinal score was assigned according to the highest documented body temperature and antipyretic requirement during the relevant assessment period: 0 = no fever (<37.5 °C), 1 = low-grade fever (37.5–37.9 °C), 2 = moderate fever (38.0–38.9 °C), and 3 = high fever (≥39.0 °C) or fever requiring repeated antipyretic use. For other symptoms, including cough, rhinorrhea, sore throat, fatigue, general condition, and physical activity level, scores were based on physician-recorded clinical notes and documented parent-reported symptom severity during routine follow-up; therefore, these assessments reflect real-world clinical documentation rather than a prospectively validated symptom scale.

Scores were derived from physician-recorded clinical notes and structured parent feedback documented during routine follow-up. Final data extraction and scoring were performed retrospectively by the study investigators based on available records.

Treatment tolerability was assessed using physician documentation and parent-reported tolerability scores on a 0–10 scale, where 0 indicated very poor tolerability and 10 indicated excellent tolerability.

Statistical analysis: Data were analyzed using SPSS version 26.0. Continuous variables are presented as mean ± standard deviation and median (interquartile range), and categorical variables as *n* (%). The distribution of continuous variables was assessed using skewness and kurtosis coefficients. For between-group comparisons, independent-samples *t* tests were used for continuous variables and chi-square or Fisher exact tests for categorical variables.

When expected cell frequencies were less than five or contingency tables contained zero counts, Fisher’s exact test was used instead of Pearson’s chi-square test, as appropriate. No formal adjustment for multiple comparisons was applied because the analyses were considered exploratory. Accordingly, *p* values should be interpreted with appropriate caution.

Changes over time and group effects were analyzed using a repeated-measures general linear model (Repeated-Measures ANOVA) including time-by-group and time-by-treatment initiation timing interactions. All tests were two-sided, and statistical significance was accepted at *p* < 0.05. Chi-square or Fisher’s exact tests were used to compare categorical symptom score distributions between groups at each individual assessment time point, whereas repeated-measures ANOVA was performed to evaluate overall within-group changes over time and time-by-group interactions. Therefore, these analyses addressed different statistical questions and should be considered complementary rather than redundant.

No multivariable regression model, propensity score matching, inverse probability weighting, or other adjusted comparative analysis was performed. Therefore, the statistical analyses should be interpreted as unadjusted exploratory comparisons.

Effect sizes and confidence intervals were not calculated for all outcomes because of the retrospective structure of the dataset and the exploratory nature of the analysis; this was acknowledged as a limitation.

No formal correction for multiple comparisons was applied because the analyses were exploratory. Therefore, statistically significant findings across multiple outcomes and time points should be interpreted cautiously due to the possibility of inflated type I error.

Potential clustering by study center was not accounted for in the statistical models, and no mixed-effects or center-adjusted model was applied.

## 3. Results

A total of 128 pediatric patients were included in the study (combined polyphenol group: *n* = 64; control group: *n* = 64). No statistically significant differences were found between the groups in terms of age, sex, anthropometric measurements, influenza type, diagnostic method, or time to treatment initiation (*p* > 0.05 for all variables). These findings indicate that the groups were homogeneous at baseline.

Baseline demographic and clinical characteristics, including sex distribution, treatment initiation timing, diagnostic method, and influenza type, were comparable between groups (Table 1).

There were also no significant differences between groups in antiviral dose (52.73 ± 8.01 vs. 53.20 ± 7.99; t(126) = −0.332, *p* = 0.741) or time to antiviral treatment initiation (3.44 ± 1.51 vs. 3.14 ± 1.32 days; t(126) = 1.197, *p* = 0.239). Skewness and kurtosis values for continuous variables were within the range of −3 to +3, indicating that the assumption of normal distribution was met for all parameters.

When changes in symptom severity scores over the follow-up period were examined, greater reductions in symptom scores were observed in all symptoms in the combined polyphenol group compared with the control group. On Day 0, there were no significant differences between groups in symptom scores (*p* > 0.05); however, by Day 3, significant differences favoring the combined polyphenol group were found in all scores for fever, cough, rhinorrhea, sore throat, fatigue, general condition, and physical activity level (*p* < 0.001 for all parameters). In the Day 5 assessment, almost complete resolution of symptoms (score 0) was observed in the combined polyphenol group, whereas symptoms persisted substantially in the control group (*p* < 0.001). On Day 7, symptom scores were recorded as zero in the combined polyphenol group, while residual symptoms, particularly fatigue and reduced physical activity level, persisted in the control group (Table 2).

Symptom score distributions across follow-up days are presented in Table 2.

When clinical recovery indicators were evaluated, the time to fever resolution was significantly shorter in the combined polyphenol group (1.84 ± 0.72 days vs. 4.50 ± 1.36 days; t(126) = −13.829, *p* < 0.001). Time to return to school was also shorter in the combined polyphenol group (3.21 ± 0.90 days vs. 5.80 ± 1.91 days; t(126) = −9.813, *p* < 0.001). Similarly, the time for mothers to return to work (2.49 ± 1.22 days vs. 6.14 ± 1.97 days; t(126) = −12.602, *p* < 0.001) and the time for fathers to return to work (1.54 ± 1.20 days vs. 5.05 ± 2.22 days; t(126) = −11.127, *p* < 0.001) were significantly shorter in the combined polyphenol group.

There were no significant differences between the combined polyphenol and control groups in antiviral dose or time to treatment initiation (52.73 ± 8.01 vs. 53.2 ± 7.99; *p* = 0.741 and 3.44 ± 1.51 vs. 3.14 ± 1.32 days; *p* = 0.239, respectively). In the combined polyphenol group, the start time was 3.44 ± 1.51 days, and the mean duration of use was 5.89 ± 1.48 days. In contrast, marked differences were observed between groups in clinical recovery parameters; the time to fever resolution (1.84 ± 0.72 vs. 4.5 ± 1.36 days), time to return to school (3.21 ± 0.9 vs. 5.8 ± 1.91 days), time for mothers to return to work (2.49 ± 1.22 vs. 6.14 ± 1.97 days), and time for fathers to return to work (1.54 ± 1.2 vs. 5.05 ± 2.22 days) were all significantly shorter in the combined polyphenol group (*p* < 0.001 for all parameters). These results suggest that combined polyphenol use was associated with better symptom control and a lower functional burden of illness, as well as potentially reduced socioeconomic impact on families. (Table 3).

Functional outcomes such as return to school and parental return to work should be interpreted with caution, as they may be influenced not only by clinical recovery but also by family circumstances, school policies, parental work flexibility, and physician or parental recommendations.

In the assessment of healthcare utilization and the need for additional treatment, the rate of additional healthcare visits on Day 3 was 20.3% in the combined polyphenol group and 53.1% in the control group (*p* < 0.001). On Day 5, no additional healthcare visits were observed in the combined polyphenol group, whereas such visits continued in 15.6% of the control group (*p* = 0.001). The need for additional antipyretic therapy was significantly lower in the combined polyphenol group, with a particularly marked difference on Days 3 and 5 (*p* < 0.001). No significant differences were found between groups in antibiotic or antitussive use (*p* > 0.05).

The need for antipyretics on Day 3 was 15.6% in the combined polyphenol group and 93.8% in the control group, representing a dramatic difference (*p* < 0.001). By Day 5, the need for antipyretics had completely disappeared in the combined polyphenol group (0%), whereas it persisted in 48.4% of the control group. These findings suggest an association between combined polyphenol use and a lower need for symptomatic medication during follow-up.

The main effect of time was significant for all symptoms (*p* < 0.001 for all parameters), and symptom scores decreased markedly throughout follow-up. The time-by-group interaction was significant for all symptoms (*p* < 0.001), indicating an association between combined polyphenol use and earlier improvement in fever, cough, rhinorrhea, sore throat, fatigue, general condition, and physical activity level scores during follow-up. In the combined polyphenol group, symptom scores generally decreased dramatically by Day 3 and disappeared completely in most parameters by Day 5, whereas recovery in the control group was slower and some symptoms, particularly fatigue and restricted physical activity, persisted until Day 7. Although the time-by-early/late treatment interaction was not significant for most symptoms, significance was observed for fatigue (*p* = 0.017), general condition (*p* = 0.004), and physical activity (*p* = 0.035), indicating that early treatment provided an additional advantage for some clinical parameters. The three-way interaction (time-by-group-by-early/late treatment) was significant only for the fever score (*p* = 0.006) and was not significant for other symptoms. These findings suggest an association between combined polyphenol use and more rapid multidimensional clinical improvement.

In the repeated-measures general linear model analysis, the main effect of time was significant for all symptom scores (*p* < 0.001), and the time-by-group interaction was significant in favor of the combined polyphenol group (*p* < 0.001). These findings suggest an association between combined polyphenol use and earlier symptom improvement during follow-up.

The effect of time on fever score was significant (*p* < 0.001), and in the total sample, the fever score decreased from 2.91 ± 0.29 on Day 0 to 1.28 ± 1.14 on Day 3, 0.47 ± 0.86 on Day 5, and 0.13 ± 0.47 on Day 7. The time-by-group interaction was significant (*p* < 0.001); in the combined polyphenol group, the fever score decreased markedly by Day 3 and disappeared completely from Day 5 onward, whereas it decreased more slowly in the control group and persisted until Day 7. Although the time-by-early/late treatment interaction was not significant (*p* = 0.119), the three-way interaction (time-by-group-by-early/late treatment) was significant (*p* = 0.006), and fever was observed to last longer particularly in late-treated patients in the control group. These findings suggest that fever resolution was observed earlier in the combined polyphenol group.

In particular, the three-way interaction was significant for the fever score (*p* = 0.006), showing that a rapid clinical response was achieved in the combined polyphenol group even among patients treated late. Similarly, the time-by-group interaction was significant across cough, rhinorrhea, general condition, and physical activity scores, supporting a potential association between combined polyphenol use and multidimensional clinical benefit.

According to physician assessment, treatment tolerability was high, with 62.5% of cases rated as “very good.” Parent-reported tolerability was also high, with a mean score of 9.39 ± 0.7 on a scale of 0 to 10.

No serious adverse events were documented in either group, and no event requiring treatment discontinuation was observed. A single participant in the combined polyphenol group experienced mild transient nausea, which resolved spontaneously without medical intervention (Table 4). No adverse events were reported in the control group. In the combined polyphenol group, the median parent-reported tolerability score was 10 (9–10), indicating excellent treatment acceptability and a high potential for adherence in clinical practice.

## 4. Discussion

This multicenter retrospective real-world data study suggests that the use of a preparation containing polyphenol, vitamin D3, vitamin C, and zinc in addition to antiviral therapy was associated with more favorable clinical recovery parameters in children diagnosed with influenza. In the present cohort, symptom scores declined more rapidly, fever resolution occurred earlier, and functional recovery measures were shorter in the combined polyphenol group than in the control group. However, because of the retrospective, observational, and non-randomized design, these findings should be interpreted as associations rather than definitive evidence of clinical efficacy. Potential residual confounding, treatment allocation bias, and unmeasured differences between groups may have contributed to the observed outcomes.

Although antiviral agents are effective in the treatment of influenza, particularly when initiated early, it is known that symptoms may be prolonged because the inflammatory response is not fully suppressed. Therefore, supportive approaches that can modulate the immune response in addition to antiviral therapy have come to the forefront in recent years. In addition to their antiviral effects, polyphenols may contribute to biological pathways of viral infections by regulating oxidative stress and cytokine responses. In vitro and clinical studies have shown that polyphenols can inhibit viral entry and replication and may shorten symptom duration in upper respiratory tract infections [8,9].

The relevance of this study to the field of pharmaceutics also lies in the evaluation of a standardized combination product rather than a single isolated compound. Polyphenol-based formulations are pharmaceutically challenging because their biological activity may be influenced by extract standardization, active compound composition, stability, and delivery characteristics. In this context, combining pomegranate peel-derived polyphenols with vitamin D3, vitamin C, and zinc may provide a formulation-based approach aimed at supporting complementary biological pathways involved in respiratory viral infections. Although the present study primarily evaluates clinical outcomes, it provides real-world data on the clinical performance and tolerability of a commercially available multi-component pharmaceutical preparation, which may be relevant for future formulation development and translational research.

Among polyphenols, pomegranate peel extract is particularly noteworthy because of its high concentrations of punicalagins, ellagic acid derivatives, and flavonoids, which confer strong antiviral and anti-inflammatory properties. The total polyphenol content of pomegranate peel has been shown to be substantially higher than that of other parts of the fruit, and these compounds may inhibit viral entry and replication by binding to viral surface proteins. In addition, pomegranate peel-derived polyphenols have been reported to modulate the host inflammatory response by suppressing nuclear factor kappa-B (NF-κB) pathways and proinflammatory cytokine responses [10,11,12].

In respiratory viral infections such as influenza, both reducing viral load and balancing the excessive inflammatory response are critically important for accelerating clinical recovery. In this context, the rapid symptom regression and early clinical response observed in our study may be associated with the multifaceted effects of highly bioactive polyphenolic compounds, particularly pomegranate peel extract. These findings may support the hypothesis that polyphenol-containing combinations could have adjunctive clinical relevance; however, causal effects cannot be inferred from the present retrospective design.

Furthermore, the effects of micronutrients such as vitamin C, vitamin D3, and zinc on the immune system are well established. Vitamin C is known to enhance the interferon response, vitamin D to regulate antimicrobial peptide expression, and zinc to play a critical role in T-cell function and mucosal immunity [13,14,15,16,17]. When used in combination, these components may theoretically support complementary immunological pathways. However, the present study cannot determine whether the observed associations were related to one specific component or to the combined formulation as a whole. The rapid symptom regression and marked shortening of recovery time observed in our study may be considered a clinical reflection of this synergistic effect.

One of the notable findings of our study was the marked decrease in symptom scores during the early period in the combined polyphenol group and the near-complete disappearance of symptoms by Day 5. The significant time-by-group interaction in repeated-measures analyses shows that the combined polyphenol group not only reduced symptom severity but also increased the speed of recovery. In addition, the marked reduction in parents’ time to return to work and in additional healthcare visits suggests that the treatment may also provide important benefits in terms of socioeconomic impact.

### Study Limitations

Several limitations of this study should be acknowledged. First, the retrospective observational design precludes causal inference and remains susceptible to residual confounding despite similar baseline characteristics between groups. Second, treatment allocation was not randomized and was determined according to routine clinical practice, introducing the possibility of treatment allocation bias and selection bias. Third, some clinical outcomes were based on parent-reported symptom assessments and recovery measures, which may be influenced by reporting inaccuracies or subjective perceptions. Fourth, no multivariable adjusted analyses, propensity score matching, or other statistical methods to control for potential confounding factors were performed. Fifth, the multicenter design may have introduced center-related variability due to differences in clinical practice patterns, patient populations, and follow-up procedures among participating institutions. Furthermore, because the investigated intervention contained multiple active components, including polyphenols, vitamin D3, vitamin C, and zinc, the individual contribution of each component to the observed associations could not be determined. In addition, virological endpoints, inflammatory biomarkers, and long-term clinical outcomes were not evaluated. Therefore, the findings should be interpreted with caution, and prospective randomized controlled studies are required to confirm these observations and clarify the specific contribution of each component.

Although the study included five centers, no formal analysis of between-center heterogeneity was performed. Therefore, it remains unclear whether the observed associations were consistent across all participating institutions.

In addition, parents who used an adjunctive preparation may have had different expectations regarding recovery, which could have influenced subjective symptom reporting.

The absence of center-adjusted or mixed-effects analyses limits the ability to evaluate site-level variability and clustering effects.

Adverse event data were not systematically collected in the retrospective database. Therefore, although no serious adverse events or treatment discontinuations were documented, quantitative comparisons of mild adverse events between groups could not be reliably performed.

The absence of comprehensive effect size estimates and confidence intervals limits the assessment of the magnitude and precision of the observed associations.

## 5. Conclusions

In this multicenter retrospective real-world dataset, the use of a polyphenol-, vitamin D3-, vitamin C-, and zinc-containing formulation in addition to antiviral therapy was associated with favorable exploratory clinical and functional outcomes in children diagnosed with influenza. However, because of the observational, non-randomized design and the possibility of residual confounding, treatment allocation bias, parent-reported outcome variability, multiplicity, and center-related heterogeneity, these findings should be interpreted cautiously. Prospective randomized controlled studies with adjusted analyses and predefined endpoints are required to confirm these observations.

Furthermore, because this was a retrospective observational study, residual confounding and treatment allocation bias cannot be completely excluded despite comparable baseline characteristics between the study groups. These methodological limitations should be considered when interpreting the observed associations.

## Figures and Tables

**Table 1:** Comparison of demographic and baseline clinical characteristics between the combined polyphenol and control groups.

		Group			*p*
		Combined Polyphenol	Control	Total	
		*n* (%)	*n* (%)	*n* (%)	
Age, years, mean ± SD		7.00 ± 1.89	7.00 ± 1.90	7.00 ± 1.89	1.000
Sex	Male	33 (51.6%)	35 (54.7%)	68 (53.1%)	0.723
	Female	31 (48.4%)	29 (45.3%)	60 (46.9%)	
Subgroup [Early treatment (≤48 h); Late treatment (>48 h)]	Early treatment	19 (29.7%)	22 (34.4%)	41 (32%)	0.705
	Late treatment	45 (70.3%)	42 (65.6%)	87 (68%)	
Diagnostic Method	Rapid test	11 (17.2%)	16 (25%)	27 (21.1%)	0.386
	PCR	53 (82.8%)	48 (75%)	101 (78.9%)	
Influenza Type	A	45 (70.3%)	45 (70.3%)	90 (70.3%)	1.000
	B	8 (12.5%)	8 (12.5%)	16 (12.5%)	
	A + B	11 (17.2%)	11 (17.2%)	22 (17.2%)	

Chi-square test.

**Table 2:** Distribution of symptom severity scores on follow-up days according to the combined polyphenol and control groups.

		Day (0)			Day (3)			Day (5)			Day (7)		
		Group		*p*	Group		*p*	Group		*p*	Group		*p*
		Combined Polyphenol	Control		Combined Polyphenol	Control		Combined Polyphenol	Control		Combined Polyphenol	Control	
		*n* (%)	*n* (%)		*n* (%)	*n* (%)		*n* (%)	*n* (%)		*n* (%)	*n* (%)	
Fever Score	0	0 (0%)	0 (0%)	0.364	49 (76.6%)	0 (0%)	<0.001	63 (100%)	31 (48.4%)	<0.001	63 (100%)	55 (85.9%)	0.003
	1	0 (0%)	0 (0%)		11 (17.2%)	4 (6.3%)		0 (0%)	10 (15.6%)		0 (0%)	2 (3.1%)	
	2	4 (6.3%)	8 (12.5%)		4 (6.3%)	40 (62.5%)		0 (0%)	19 (29.7%)		0 (0%)	7 (10.9%)	
	3	60 (93.8%)	56 (87.5%)		0 (0%)	20 (31.3%)		0 (0%)	4 (6.3%)		0 (0%)	0 (0%)	
Cough Score	0	1 (1.6%)	0 (0%)	0.424	45 (70.3%)	3 (4.7%)	<0.001	63 (100%)	38 (59.4%)	<0.001	63 (100%)	55 (85.9%)	0.003
	1	0 (0%)	0 (0%)		16 (25%)	4 (6.3%)		0 (0%)	7 (10.9%)		0 (0%)	4 (6.3%)	
	2	6 (9.4%)	10 (15.6%)		3 (4.7%)	40 (62.5%)		0 (0%)	16 (25%)		0 (0%)	5 (7.8%)	
	3	57 (89.1%)	54(%8.,4)		0(%0)	17 (26.6%)		0 (0%)	3 (4.7%)		0 (0%)	0 (0%)	
Rhinorrhea Score	0	2 (3.1%)	0 (0%)	0.499	46 (71.9%)	6 (9.4%)	<0.001	63 (100%)	43 (67.2%)	<0.001	63 (100%)	56 (87.5%)	0.006
	1	0 (0%)	0 (0%)		16 (25%)	5 (7.8%)		0 (0%)	7 (10.9%)		0 (0%)	6 (9.4%)	
	2	8 (12.5%)	10 (15.6%)		2 (3.1%)	40 (62.5%)		0 (0%)	13 (20.3%)		0 (0%)	2 (3.1%)	
	3	54 (84.4%)	54 (84.4%)		0 (0%)	13 (20.3%)		0 (0%)	1 (1.6%)		0 (0%)	0 (0%)	
Sore Throat Score	0	0 (0%)	0 (0%)	1.000	50 (78.1%)	6 (9.4%)	<0.001	63 (100%)	45 (70.3%)	<0.001	63 (100%)	60 (93.8%)	0.119
	1	0 (0%)	0 (0%)		12 (18.8%)	4 (6.3%)		0 (0%)	6 (9.4%)		0 (0%)	2 (3.1%)	
	2	9 (14.1%)	10 (15.6%)		2 (3.1%)	47 (73.4%)		0 (0%)	12 (18.8%)		0 (0%)	2 (3.1%)	
	3	55 (85.9%)	54 (84.4%)		0 (0%)	7 (10.9%)		0 (0%)	1 (1.6%)		0 (0%)	0 (0%)	
Fatigue Score	0	0 (0%)	0 (0%)	1.000	28 (44.4%)	2 (3.1%)	<0.001	62 (98.4%)	3 (4.7%)	<0.001	63 (100%)	18 (28.1%)	<0.001
	1	0 (0%)	0 (0%)		28 (44.4%)	4 (6.3%)		1 (1.6%)	6 (9.4%)		0 (0%)	5 (7.8%)	
	2	12 (18.8%)	13 (20.3%)		7 (11.1%)	53 (82.8%)		0 (0%)	52 (81.3%)		0 (0%)	41 (64.1%)	
	3	52 (81.3%)	51 (79.7%)		0 (0%)	5 (7.8%)		0 (0%)	3 (4.7%)		0 (0%)	0 (0%)	
General Condition Score	0	0 (0%)	0 (0%)	1.000	53 (82.8%)	8 (12.5%)	<0.001	63 (100%)	39 (60.9%)	<0.001	63 (100%)	54 (84.4%)	0.002
	1	0 (0%)	0 (0%)		11 (17.2%)	7 (10.9%)		0 (0%)	12 (18.8%)		0 (0%)	9 (14.1%)	
	2	18 (28.1%)	18 (28.1%)		0 (0%)	48 (75%)		0 (0%)	13 (20.3%)		0 (0%)	1 (1.6%)	
	3	46 (7.,9%)	46 (71.9%)		0 (0%)	1 (1.6%)		0 (0%)	0 (0)		0 (0%)	0 (0%)	
Physical Activity Level Score	0	0 (0%)	0 (0%)	0.692	51 (79.7%)	9 (14.1%)	<0.001	63 (100%)	41 (64.1%)	<0.001	63 (100%)	52 (81.3%)	<0.001
	1	1 (1.6%)	0 (0%)		13 (20.3%)	9 (14.1%)		0 (0%)	15 (23.4%)		0 (0%)	11 (17.2%)	
	2	16 (25%)	19 (29.7%)		0 (0%)	46 (71.9%)		0 (0%)	8 (12.5%)		0 (0%)	1 (1.6%)	
	3	47 (73.4%)	45 (70.3%)		0 (0%)	0 (0%)		0 (0%)	0 (0%)		0 (0%)	0 (0%)	

Pearson’s chi-square test or Fisher’s exact test, as appropriate.

**Table 3:** Comparison of treatment characteristics and recovery process indicators between the combined polyphenol and control groups.

	Group						*p*
	Combined Polyphenol		Control		Total		
	Mean ± SD	Median (Q25-Q75)	Mean ± SD	Median (Q25-Q75)	Mean ± SD	Median (Q25-Q75)	
Antiviral Dose	52.73 ± 8.01	52.5 (45–75)	53.2 ± 7.99	60 (45–75)	52.97 ± 7.97	60 (45–75)	0.741
Antiviral Start Time (day)	3.44 ± 1.51	3 (1–7)	3.14 ± 1.32	3 (1–5)	3.29 ± 1.42	3 (1–7)	0.239
Combined Polyphenol Start Time (day)	3.44 ± 1.51	3 (1–7)					
Duration of Combined Polyphenol Use	5.89 ± 1.48	5 (5–10)					
Time to Fever Resolution (day)	1.84 ± 0.72	2 (1–3)	4.5 ± 1.36	5 (3–7)	3.18 ± 1.72	3 (1–7)	<0.001
Time to Return to School (day)	3.21 ± 0.9	3 (2–5)	5.8 ± 1.91	5 (3–10)	4.51 ± 1.98	5 (2–10)	<0.001
Time for Mother to Return to Work (day)	2.49 ± 1.22	3 (0–5)	6.14 ± 1.97	5 (3–10)	4.33 ± 2.46	5 (0–10)	<0.001
Time for Father to Return to Work (day)	1.54 ± 1.2	1 (0–5)	5.05 ± 2.22	5 (0–10)	3.31 ± 2.51	3 (0–10)	<0.001

Independent-samples *t* test. Values are presented as mean ± SD and median (Q25–Q75).

**Table 4:** Summary of safety outcomes and treatment tolerability in the combined polyphenol and control groups.

Safety Outcome	Combined Polyphenol (*n* = 64)	Control (*n* = 64)	*p*-Value
Any adverse event, *n* (%)	1 (1.6%)	1 (1.6%)	1.000
Type of adverse event	Mild transient nausea	Mild transient nausea	—
Serious adverse event, *n* (%)	0 (0%)	0 (0%)	—
Treatment discontinuation due to AE, *n* (%)	0 (0%)	0 (0%)	—

## Data Availability

The original contributions presented in this study are included in the article. Further inquiries can be directed to the corresponding author.

## References

[1] Troeger C.E., Blacker B.F., Khalil I.A., Zimsen S.R., Albertson S.B., Abate D., Abdela J., Adhikari T.B., Aghayan S.A., Agrawal S. (2019). Mortality, morbidity, and hospitalisations due to influenza lower respiratory tract infections, 2017: An analysis for the Global Burden of Disease Study 2017. Lancet Respir. Med..

[2] Lafond K.E., Porter R.M., Whaley M.J., Suizan Z., Ran Z., Aleem M.A., Thapa B., Sar B., Proschle V.S., Peng Z. (2021). Global burden of influenza-associated lower respiratory tract infections and hospitalizations among adults: A systematic review and meta-analysis. PLoS Med..

[3] Malosh R.E., Martin E.T., Heikkinen T., Brooks W.A., Whitley R.J., Monto A.S. (2018). Efficacy and safety of oseltamivir in children: Systematic review and individual patient data meta-analysis of randomized controlled trials. Clin. Infect. Dis..

[4] Muthuri S.G., Venkatesan S., Myles P.R., Leonardi-Bee J., Al Khuwaitir T.S., Al Mamun A., Anovadiya A.P., Azziz-Baumgartner E., Báez C., Bassetti M. (2014). Effectiveness of neuraminidase inhibitors in reducing mortality in patients admitted to hospital with influenza A H1N1pdm09 virus infection: A meta-analysis of individual participant data. Lancet Respir. Med..

[5] Calder P.C., Carr A.C., Gombart A.F., Eggersdorfer M. (2020). Optimal Nutritional Status for a Well-Functioning Immune System Is an Important Factor to Protect against Viral Infections. Nutrients.

[6] Yahfoufi N., Alsadi N., Jambi M., Matar C. (2018). The Immunomodulatory and Anti-Inflammatory Role of Polyphenols. Nutrients.

[7] Ding S., Jiang H., Fang J. (2018). Regulation of Immune Function by Polyphenols. J. Immunol. Res..

[8] Chojnacka K., Skrzypczak D., Mikula K., Witek-Krowiak A., Izydorczyk G. (2021). Antiviral Properties of Polyphenols from Plants. Foods.

[9] Ninfali P., Antonelli A., Magnani M., Scarpa E.S. (2020). Antiviral Properties of Flavonoids and Delivery Strategies. Nutrients.

[10] Viuda-Martos M., Fernández-López J., Pérez-Álvarez J.A. (2010). Pomegranate and its many functional components as related to human health: A review. Compr. Rev. Food Sci. Food Saf..

[11] Tito A., Colantuono A., Pirone L., Pedone E., Intartaglia D., Giamundo G., Conte I., Vitaglione P., Apone F. (2021). Pomegranate Peel Extract as an Inhibitor of SARS-CoV-2 Spike Binding to Human ACE2 Receptor in vitro: A Promising Source of Novel Antiviral Drugs. Front. Chem..

[12] Ismail T., Sestili P., Akhtar S. (2012). Pomegranate peel and fruit extracts: A review of potential anti-inflammatory and anti-infective effects. J. Ethnopharmacol..

[13] Arıca S., Arıca V., Dag H., Kaya A., Hatipoglu S., Fenercioglu A., Karatekin G. (2011). Serum Zinc levels in children of 0-24 months diagnosed with pneumonia admitted to our clinic. Int. J. Clin. Exp. Med..

[14] Silfeler I., Tanidir I.C., Arica V. (2012). Risk factors for lower respiratory tract infections in children. Pak. J. Med. Sci. Q..

[15] Hemilä H. (2017). Vitamin C and infections. Nutrients.

[16] Martineau A.R., Jolliffe D.A., Hooper R.L., Greenberg L., Aloia J.F., Bergman P., Dubnov-Raz G., Esposito S., Ganmaa D., Ginde A.A. (2017). Vitamin D supplementation to prevent acute respiratory infections. BMJ.

[17] Read S.A., Obeid S., Ahlenstiel C., Ahlenstiel G. (2019). The Role of Zinc in Antiviral Immunity. Adv. Nutr..

